# Thiourea and hydrogen peroxide priming improved K^+^ retention and source-sink relationship for mitigating salt stress in rice

**DOI:** 10.1038/s41598-020-80419-6

**Published:** 2021-02-04

**Authors:** Manish Pandey, Radha Krishna Paladi, Ashish Kumar Srivastava, Penna Suprasanna

**Affiliations:** 1 Nuclear Agriculture and Biotechnology Division, Bhabha Atomic Research Centre, Mumbai, 400085 India; 2 Homi Bhabha National Institute, Mumbai, 400094 India

**Keywords:** Plant sciences, Plant stress responses, Salt

## Abstract

Plant bioregulators (PBRs) represent low-cost chemicals for boosting plant defense, especially under stress conditions. In the present study, redox based PBRs such as thiourea (TU; a non-physiological thiol-based ROS scavenger) and hydrogen peroxide (H_2_O_2_; a prevalent biological ROS) were assessed for their ability to mitigate NaCl stress in rice variety IR 64. Despite their contrasting redox chemistry, TU or H_2_O_2_ supplementation under NaCl [NaCl + TU (NT) or NaCl + H_2_O_2_ (NH)] generated a reducing redox environment *in planta*, which improved the plant growth compared with those of NaCl alone treatment. This was concomitant with better K^+^ retention and upregulated expression of NaCl defense related genes including *HAK21*, *LEA1*, *TSPO* and *EN20* in both NT and NH treated seedlings. Under field conditions, foliar applications of TU and H_2_O_2_, at vegetative growth, pre-flowering and grain filling stages, increased growth and yield attributes under both control and NaCl stress conditions. Principal component analysis revealed glutathione reductase dependent reduced ROS accumulation in source (flag leaves) and sucrose synthase mediated sucrose catabolism in sink (developing inflorescence), as the key variables associated with NT and NH mediated effects, respectively. In addition, photosystem-II efficiency, K^+^ retention and source-sink relationship were also improved in TU and H_2_O_2_ treated plants. Taken together, our study highlights that reducing redox environment acts as a central regulator of plant’s tolerance responses to salt stress. In addition, TU and H_2_O_2_ are proposed as potential redox-based PBRs for boosting rice productivity under the realistic field conditions.

## Introduction

The rapid increase in the population together with shrinking of cultivable land is one of the major challenges towards ensuring global food security^1^. Salinity stress affects ~ 20% of world’s irrigated land and can go upto 50% of cultivable land by the middle of the twenty-first century^2^. Salinity decreases the soil water potential and thereby imposes an osmotic barrier for water uptake. Moreover, increased Na^+^ uptake also induces ionic toxicity which adversely affects overall plant metabolism^3^. Both osmotic and ionic disequilibrium results in increased production of reactive oxygen species (ROS), causing oxidative damage to various biomolecules like protein, lipid and DNA^4^. Plants possess various defense mechanisms including the activation of antioxidant system, maintenance of ionic homeostasis and activation of the defense genes. The non-enzymatic (phenolic, flavonoids, tocopherol, carotenoids, ascorbate (ASC), and glutathione (GSH) and enzymatic (superoxide dismutase, catalase, guaiacol peroxidase, ascorbate peroxidase, glutathione reductase) antioxidants functions to control the level of ROS^5^. Further the sequestration/redistribution of Na^+^ ions under salt stress maintains K^+^/Na^+^ homeostasis and thereby suppresses metabolic arrest and growth impairment^6^. Besides this, the activation of defense associated genes like late embryogenic abundant proteins (LEA)/dehydrin^7^, K^+^ transporters^8^, signalling mediators like phosphatidylinositol-4-phosphate 5 kinase^9^ (*PIPK*), translocator protein^10^ (TSPO) and early nodulin-20^11^ (EN20) are also reported to boost the ameliorative potential of plant towards salt stress.

Both osmotic and ionic components of the salt stress negatively affect plant growth and yield associated traits^12^. Various physiological processes like cell expansion, stomatal development, movement and conductance of leaf are negatively affected under salt stress^13^. In most plant systems, decrease in net photosynthetic rate^14^ was reported as one of the earliest quantifiable responses under salt stress. In plant photosynthesis, carbon dioxide (CO_2_) is fixed in chloroplasts via the Calvin cycle to yield triose phosphates (triose-P). Triose-P is transported to cytosol by triose-P/phosphate transporter for the synthesis of sucrose. In cytosol, aldolases catalyse the formation of fructose 1,6-bisphosphate (F1,6BP), which is further metabolized to yield sucrose by the combined action of fructose 1,6-bisphosphatase and sucrose phosphate synthase (SPS). Sucrose as a final product of photo assimilation is translocated from its synthesis site (leaf) to various non-photosynthetic tissues^15^ (sink tissues) via phloem tissue. Sucrose translocated to sink is metabolized by cytosolic neutral invertase (NI), sucrose synthase (SuSy), and vacuolar acid invertase (AI), providing the hexose pool for the synthesis of structural and non-structural carbohydrates. The invertases (INV) catalyse irreversible hydrolyzation of sucrose into its hexose monomers (glucose and fructose), whereas SuSy catalyses reversible cleavage of sucrose using UDP to yield fructose and UDP-G. Salt stress negatively affects the sugar dynamics of the source leaf and developing sink tissue by inhibiting the synthesis, redistribution and utilisation of sucrose^16^.

Rice is the staple food crop which feeds more than half of the world population. Among the different abiotic stresses, soil salinity poses a major constraint for rice productivity. For rice, critical salinity level is estimated to be 6.9 dS m^−1^ which leads to 50% yield loss^17^. Salt sensitivity also varies with age, from being moderately tolerant at seedling stage to highly susceptible at reproductive phase^18^. External application of chemicals and bio-molecules, referred to as plant bioregulators (PBRs), has been shown to minimize the salt-stress induced yield losses in multiple crops. Though PBRs may act differently, a unified redox/ROS dependent action has recently been proposed^19^. In the present study, two redox based PBRs such as thiourea^20^ (TU, non-physiological thiol-based ROS scavenger) and H_2_O_2_^21,22^ (ROS signalling molecule), which chemically drive ROS levels in opposite direction, were assessed whether they will have an overlapping or independent effect(s). To this end, a short-term study was conducted at seedling stage to understand the impact of nutrient medium-supplemented TU and H_2_O_2_ on redox and ionic equilibrium. Further, the effects of foliar supplemented TU and H_2_O_2_ on field-grown plants were also studied in terms of source-sink relationship and yield attributes. The findings revealed that both TU and H_2_O_2_ maintain reduced redox status that could act as “core” regulator for improving K^+^ retention ability, photosynthetic efficiency and source-sink strength of the plants. These changes were ultimately reflected in the form of improved growth and yield under both control and NaCl stress conditions.

## Materials and methods

### Plant material, growth conditions and stress treatment

The study was performed on Indian rice (*O. sativa*) var. IR-64. The seeds were surface sterilized using 30% ethanol for 3 min followed by repeated washing with distilled water to remove traces of ethanol. The surface-sterilized seeds were germinated for 48 h and hydroponic cultures were established, as per the method described previously^23^. The ameliorative potential of TU and H_2_O_2_ towards NaCl stress was evaluated using two independent approaches. In the first approach, 14 days old hydroponically grown seedlings were subjected to different treatments including control (Yoshida medium), NaCl (50 mM), TU (7.5 µM), NaCl (50 mM) + TU (7.5 µM), H_2_O_2_ (1 µM) and NaCl (50 mM) + H_2_O_2_ (1 µM). Hereafter, NaCl + TU and NaCl + H_2_O_2_ treatments were denoted as NT and NH, respectively. A pre-treatment of 7.5 µM TU (TU and NT) and 1 µM H_2_O_2_ (H_2_O_2_ and NH) was also given for 24 h. The pre-treatment strategy has already been demonstrated to maximize the impact of TU-mediated amelioration of AsV in rice^23^. In shoots, activities of antioxidant enzyme and Na^+^ and K^+^ accumulation were quantified in a time-course manner ranging from 1, 4, 24 and 48 h post-stress. In addition, 6 h after the onset of treatments, expression levels of selected salt-responsive genes were analyzed. At 7 days post-stress, phenotypic parameters, both qualitative and quantitative along with antioxidant capacity were recorded. In the second approach, four healthy 30 days old hydroponically grown seedlings were transferred to plastic pots in six groups (5 pots/group), under the net-house experimental facility of Bhabha Atomic Research Centre, Mumbai (India). The fertigation and agronomic protocols were followed as previously described^24^. Group-1 plants were treated with NaCl (11 g/pot) dose twice, at 42- and 57-days post-transplantation. The NaCl dose (22 g NaCl/per pot; equivalent to ~ 62 mM) was calculated considering the total water holding capacity of 14 kg paddy soil (4.6 L) and the top-water (1.4 L). The group-2 and -3 plants were given foliar applications of TU (6.5 mM containing 0.01% Tween-20) and H_2_O_2_ (1 mM containing 0.01% Tween-20), respectively. A total of three foliar applications were given at vegetative, early anthesis and grain filling stages that corresponded to 40, 55 and 72 days post-transplantation, respectively. Group-4 and -5 plants were given the combined treatment of NaCl + TU (NT) and NaCl + H_2_O_2_ (NH) treatments, respectively. Group-6 plants were foliar-sprayed with water (three times at 40, 55 and 72 days post-transplantation) and served as control. At 5 days post 3rd-foliar spray, various morphological traits (plant height, flag leaf length and width, tiller number, panicle number and length, chlorophyll content) were recorded and biochemical attributes (superoxide radical imaging, GR activity, ASA/DHA ratio and photosynthetic efficiency) were quantified from the flag leaves. Additionally, parameters of plant source-sink relationship and ion accumulation were quantified in three different tissues such as youngest flag leaf, old leaf from the bottom and developing inflorescence.

### Measurement of antioxidant capacity and activities of antioxidant enzymes

The non-enzymatic antioxidant status of leaf tissues was analyzed according to the Oxygen Radical Absorbance Capacity (ORAC) method^25^. For the measurement of antioxidant enzyme activities, total protein was extracted from liquid N_2_ ground plant material (~ 250 mg) using the pre-chilled buffer [(was extracted using 100 mM chilled potassium phosphate buffer (pH 7.0) containing 0.1 mM EDTA and 1% polyvinyl pyrrolidone (w/v)]. The samples were centrifuged at 15,000×*g* for 15 min at 4 °C. The supernatant was separated and used for the measurement of superoxide dismutase (SOD), catalase (CAT), ascorbate peroxidase (APX) and glutathione reductase (GR) activities^26^. The protein content in the supernatant was quantified as per Bradford method^27^.

### Ion content analysis

For the quantification of ions, leaf samples were dried at 70 °C till they achieve constant weight. The dried tissues (~ 100 mg) were acid digested using concentrated HNO_3_, and finally dissolved using Milli-Q water. The Na^+^ and K^+^ contents were quantified using atomic absorption spectrometer and represented as % dry weight (DW).

### RNA isolation and real-time expression profiling

The leaf tissue samples from various treatments were harvested under liquid N_2_ and subjected to total RNA isolation using TRI-reagent (Sigma T-9494), as per manufacturer’s instructions. The quality assessment of the RNA sample and subsequent cDNA preparation were performed as described previously^23^. The details of gene-specific primers are given as Supplementary Table 1.

### Quantification of plant growth and yield parameters

The various plant growth parameters viz shoot length, flag leaf length, leaf width and panicle length were quantified using a meter scale. The number of panicles per plant and 1000 seed weight were quantified manually. The leaf chlorophyll content was calculated as SPAD value using Chlorophyll Meter SPAD-502 plus-konica Minolta, representing an average value of five different points in the same leaf.

### Measurement of stomatal conductance and PS-II stability

The gas exchange of leaves was measured using an Infrared Gas Analyzer, GFS-3000 (Walz, Germany). The photosynthetic photon flux density (PPFD) was fixed at 1000 μmol m^−2^ s^−1^ after optimization with a light curve. The photosynthetic efficiency was quantified using following parameters; cuvette air flow 750 mL min^−1^, chamber temperature (25 °C), Relative humidity (60%) and atmospheric CO_2_ concentration (400 ppm CO_2_). Net photosynthetic rate, stomatal conductance, and transpiration were recorded simultaneously. Water use efficiency (WUE) was calculated as the ratio between net photosynthesis and transpiration. Using the differential minimum (F_o_) and maximum fluorescence (F_m_) signals of from the open and close PS II centers, maximum quantum efficiency of PSII (F_v_/F_m_) was quantified. A 20 min prior dark adaptation is a prerequisite for the quantification of F_v_/F_m,_ and can be defined as (F_m_ − F_o_)/F_m_. Similarly, maximum fluorescence of dark (F_m_)and light adapted leaf (F_m’_) were used for the quantification of Non photochemical quenching (NPQ); NPQ = Fm/F′m − 1. Further steady state chlorophyll fluorescence (Fs) was also quantified for the measurement of ETR and actual quantum efficiency of PSII (Φ_PSII_); ΦPSII = (F′m − Fs)/F′m^28^.

### Histochemical detection of superoxide radical and quantification of ascorbate pool

Superoxide radicals were detected in situ using nitroblue tetrazolium (NBT) staining^29^. NBT stain intensity was quantified using image J software (version 1.53d; https://imagej.nih.gov). The levels of ascorbate (ASA) and dehydroascorbate (DHA) contents were quantified using α-α′-bipyridyl-based colorimetric method^30^ and the results were presented as ASA/DHA ratio.

### Quantification of sucrose and starch levels

Lyophilized leaf sample (10 mg) was extracted in 15 mL of 80% ethanol. The extract was boiled for 10 min and then subjected to centrifugation at 15,000*g* for 15 min at room temperature. The sucrose and starch were quantified in the supernatant and pellet respectively using sucrose quantification kit (SCA-20; Sigma) and starch assay kit (STA-20; Sigma Aldrich), respectively according to the manufacturer's protocol.

### Measurement of the activities of source-sink homeostasis related enzymes

For the quantification of enzymatic activities related to source sink homeostasis, total protein was extracted from liquid N_2_ ground plant material (~ 300 mg) using the pre-chilled buffer [(containing 100 mM chilled MOPS (50 mM; pH 7.5), MgCl_2_ (15 mM), EDTA (1 mM), poly-vinylpyrrolidone (2%; w/v), and phenyl methyl sulfonyl fluoride (2 mM)]. The samples were centrifuged at 12,000×*g* for 15 min at 4 °C. The supernatant was separated and used for the measurement of SPS, SuSy^31^ and FPBase^32^. Additionally, Neutral invertase (NI) and acid soluble invertase (AI) activity in the plant samples were measured spectrophometrically following the extraction and assay methods^31^. The protein content in the sample was measured as per the Bradford method. All the enzyme activities were represented as units mg^−1^ protein which corresponds to μM of the product formed mg^−1^ protein min^−1^.

### Statistical analysis

The experiments were conducted as randomized block design using three biological replicates. One-way analysis of variance (ANOVA) was performed with the whole dataset to confirm the variability of data and validity of results, and Duncan’s multiple range test (DMRT) was performed to determine the significant difference between treatments. Different letters indicate significantly different values (DMRT, *p* ≤ 0.05). Principal component analysis (PCA) was performed with datasets of the source leaf and developing sink, using Origin 2016 (Origin Lab, Northampton, MA, USA), and the first two components (PC1 and PC2) explaining the maximum variance in the datasets were used to make biplots.

## Results

### TU and H_2_O_2_ ameliorate NaCl stress through enhanced antioxidant capacity

**Figure 1:**
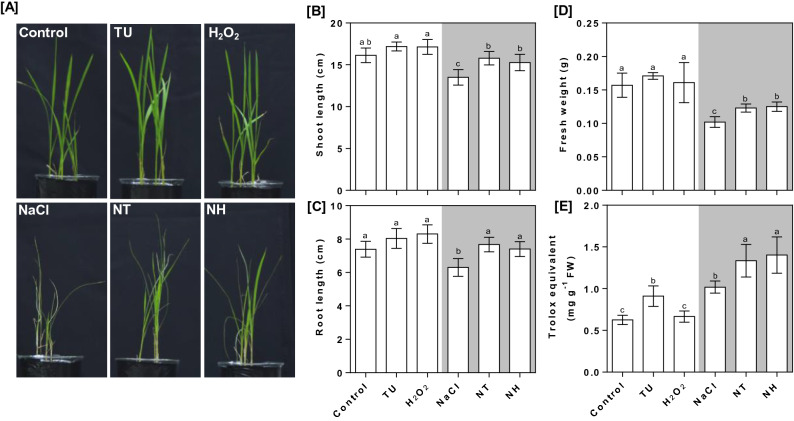
Post-germination phenotyping of rice seedlings under different treatments. The rice seedlings were grown hydroponically for 14 days under control conditions and then, subjected to different treatments including control (Yoshida medium), NaCl (50 mM), TU (7.5 µM), NaCl (50 mM) + TU (7.5 µM) [NT], H_2_O_2_ (1 µM) and NaCl (50 mM) + H_2_O_2_ (1 µM) [NH]. After 7 days of treatment, differential phenotyping was observed qualitatively (**A**) and also quantified in terms of shoot length (**B**), root length (**C**), seedlings fresh weight (**D**). In addition, the total antioxidant capacity as trolox equivalent was also quantified from shoot part (**E**). For NT and NH, 24 h pretreatment of 7.5 µM TU and 1 µM H_2_O_2_, respectively was also given. All the values are mean of triplicates ± SD. Different letters indicate significantly different values (DMRT, *p* ≤ 0.05).

There was a considerable growth reduction in terms of leaf drying and fragile stem in the NaCl-treated seedlings (Fig. 1A). Both root and shoot lengths were decreased by 14.75 and 16.36% (Fig. 1B,C) and fresh biomass was reduced by 35.03% (Fig. 1D) in NaCl-treated seedlings compared with that of control. Significant growth restoration was observed in NT and NH treatments respectively, in terms of shoot length (16.96 and 13.11%; Fig. 1B), root length (21.75 and 17.46%; Fig. 1C), and biomass (20.59 and 22.55%; Fig. 1D), compared to NaCl-treated seedlings. No significant change in growth attributes was observed under TU and H_2_O_2_ alone treatments. The total antioxidant capacity was increased by 62.80% in NaCl-treated seedlings compared with that of control. An additional increase of 31.05 and 37.7% was observed under NT and NH, respectively, compared with those of NaCl treatment (Fig. 1E). Although no significant change in antioxidant capacity was observed in H_2_O_2_-treated seedlings; however, under TU alone treatment, it was increased by 45.56% over control.

### Temporal kinetics of antioxidant enzyme activities and ion accumulations in shoot

**Figure 2:**
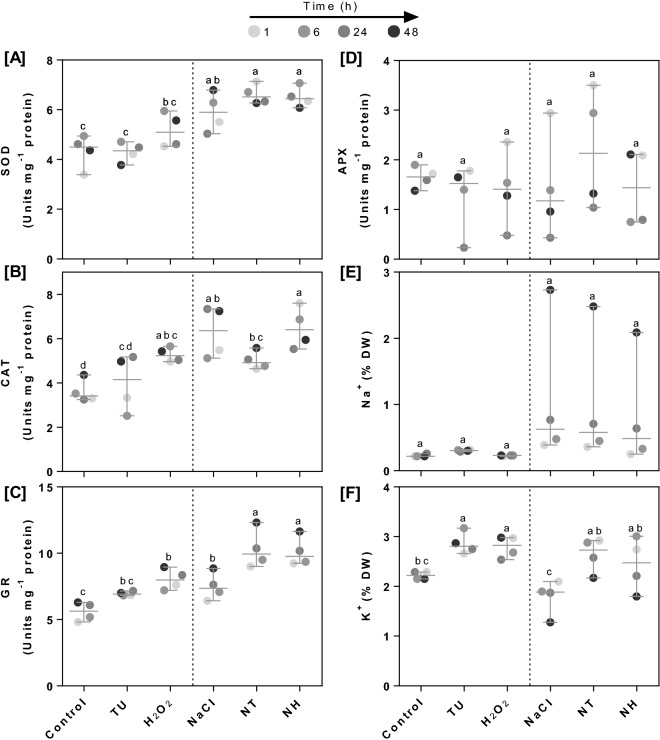
The temporal regulation of antioxidant enzyme activities and levels of Na^+^ and K^+^ in rice seedlings. The rice seedlings were grown hydroponically for 14 days under control conditions and then, subjected to different treatments including control (Yoshida medium), NaCl (50 mM), TU (7.5 µM), NaCl (50 mM) + TU (7.5 µM) [NT], H_2_O_2_ (1 µM) and NaCl (50 mM) + H_2_O_2_ (1 µM) [NH]. After 1, 6, 24 and 48 h of treatment, the leaf tissue was harvested and analyzed for superoxide dismutase (SOD; **A**), catalase (CAT; **B**), gutathione reductase (GR; **C**) and ascorbate peroxidase (APX; **D**), Na^+^ (**E**) and K^+^ (**F**). For NT and NH, 24 h pretreatment of 7.5 µM TU and 1 µM H_2_O_2_ respectively was also given. The data represented in the form of median value with range was computed using individual data from all four time-points (1, 6, 24 and 48 h). Refer supplementary table 2 for statistics of individual time point. Different letters indicate significantly different values (DMRT, *p* ≤ 0.05).

The temporal regulation of antioxidant enzyme activities and levels of Na^+^ and K^+^ were assessed in leaves subjected to different treatments. In order to understand the overall pattern, median value was computed using individual data from all four time-points (1, 6, 24 and 48 h) and compared across different treatments (Fig. 2; Supplementary table 2). Under NaCl treatment, SOD, CAT and GR activities were increased by 31.15, 86.33 and 30.49%, respectively compared with those of control. While SOD and GR activities were further increased by (10.6 and 35.19%) and (9.25 and 32.92%) under NT and NH treatments respectively, CAT activity was decreased by 22.72% in NT treatment, compared with those under NaCl treatment (Fig. 2A–C). Additionally, SOD, CAT and GR activities were also increased under TU and H_2_O_2_ alone treatments as compared with those of control; except for SOD activity in TU-treated leaves. In general, increase in antioxidant enzyme activities was higher in H_2_O_2_ than TU alone treatment (Fig. 2A–C). No significant change in median of APX activity was observed under any treatment conditions (Fig. 2D). However, at 48 h time point, APX activity was significantly reduced by 29% in NaCl-treated leaves as compared with control. Besides, under NT and NH treatments, APX was increased by 81.28 and 22.55%, respectively as compared with that of NaCl treatment. At the same time-point, no significant change in APX activity was noticed under TU and H_2_O_2_ alone treatments (Supplementary table 2D). The overall pattern of Na^+^ accumulation remained unchanged across various treatments (Fig. 2E); however, better K^+^ retention was observed under TU and H_2_O_2_ alone as well as NT and NH treatments (Fig. 2F). At 48 h, upto 59.22% decrease in K^+^ accumulation was observed under NaCl as compared to control. At the same time point (48 h), K^+^ accumulation was increased by 147.32 and 105.47% under NT and NH treatments, respectively compared to NaCl treatment. Additionally, 33.42, 38.58% increase under TU, H_2_O_2_ was also observed at 48 h compared to control (Supplementary table 2F).

### Expression levels of early NaCl stress responsive genes under different treatments

**Figure 3:**
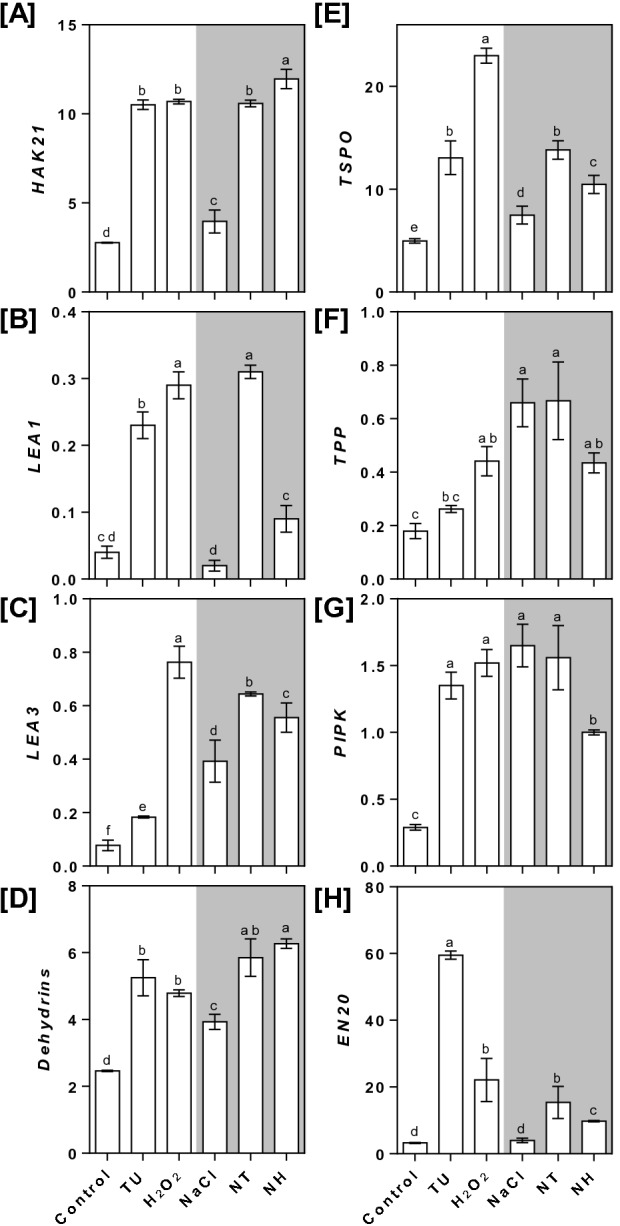
Real-time PCR based quantification of NaCl stress responsive genes in rice seedlings under different treatments. The rice seedlings were grown hydroponically for 14 days under control conditions and then, subjected to different treatments including control (Yoshida medium), NaCl (50 mM), TU (7.5 µM), NaCl (50 mM) + TU (7.5 µM) [NT], H_2_O_2_ (1 µM) and NaCl (50 mM) + H_2_O_2_ (1 µM) [NH]. After 6 h of treatment, the leaf tissue was harvested and analysed for relative expression of high‐affinity K^+^ transporter (*HAK21*; **A**), late embryogenic abundant protein-1 (*LEA1*; **B**), late embryogenic abundant protein-3 (LEA3; **C**), dehydrin (**D**), translocator protein (*TSPO*; **E**), trehalose-phosphate phosphatase (*TPP*; **E**), phosphatidylinositol-4-phosphate 5 kinase (*PIPK*; **F**) and early nodulin-20 (*EN20*; **H**). All the values are mean of triplicates ± SD and are normalized using *tubulin* as constitutive gene. Different letters indicate significantly different values (DMRT, *p* ≤ 0.05*)*.

The top-ranked eight early NaCl stress responsive genes were selected on the basis of published transcriptome of rice seedlings^33^ and their expression levels were measured in the shoot tissue under different treatments. The results revealed that both TU and H_2_O_2_ treatments led to upregulated expression under control as well as NaCl stress conditions (Fig. 3). The major effects were seen in *HAK21* (high‐affinity K^+^ transporter), *LEA1* (late-embryogenic abundant 1), *dehydrin* and *TSPO* (translocator protein) which were upregulated by 279.42, 475, 113.41 and 162.78% under TU, and 285.92, 625, 94.72 and 362.58% under H_2_O_2_-treated leaves compared to control. Similarly, the expression of *HAK21* (166.75%; NT and 201.42; NH), *LEA1* (1450%; NT and 350%; NH), *dehydrin* (48.85%; NT and 59.54%; NH) and *TSPO* (84.89%; NT and 39.97%; NH) was found to be upregulated compared with those of NaCl-treated leaves (Fig. 3A–D). Other tested genes *TPP*, *PIPK* and *EN20* were mainly upregulated under TU and H_2_O_2_ alone treatments compared with those of control. Under NT treatment, the expression of these genes remained unchanged except for *EN20* which was upregulated by 285.06% compared with those of NaCl treatment. In NH-treated leaves, *TPP* and *PIPK* were downregulated by 34.08 and 39.39%, while *EN20* was upregulated by 144.67% compared with those of NaCl treatment (Fig. 3F–H).

### Foliar supplementation of TU and H_2_O_2_ enhanced plant growth and yield attributes

**Figure 4:**
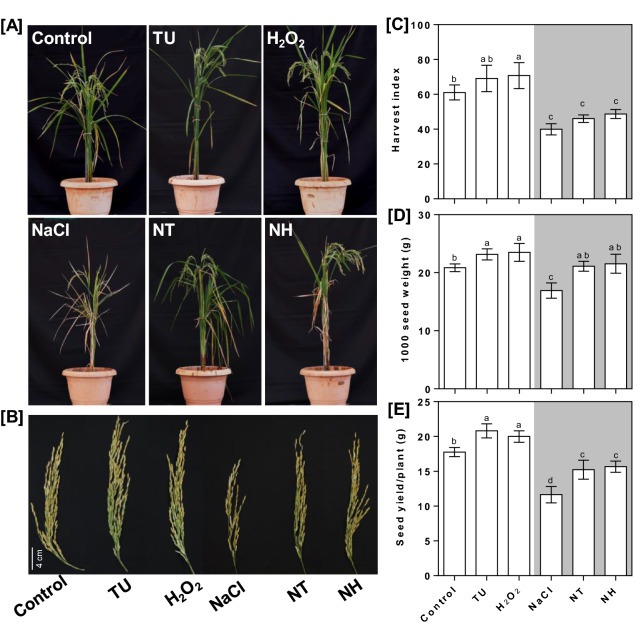
Effect of foliar-applied TU and H_2_O_2_ treatment on plant growth and yield attributes. Pot-studies were performed under different treatments including WS (DW + 0.01% Tween-20), TU (6.5 mM TU + 0.01% Tween-20) and H_2_O_2_ (10 mM H_2_O_2_ + 0.01% Tween-20), NaCl (22 g NaCl per pot). NT and NH denote combined treatment of NaCl + TU and NaCl + H_2_O_2_ treatments, respectively. A total of three foliar applications were given at vegetative, early anthesis and grain filling stages that corresponded to 40, 55 and 72 days post-transplantation, respectively. At the time of maturity, the differential phenotype was recorded (**A**) and representative panicles were shown (**B**). In addition, the yield parameters such as harvest index (**C**), seed yield per plant (**D**) and 1000 seed weight (**E**) were also quantified. All values represent mean of 10 plants ± S.E. Different letters indicate significantly different values (DMRT, *p* ≤ 0.05).

The foliar application of TU and H_2_O_2_ showed improvement in plant growth and yield attributes at mature plant stage (Fig. 4A). The major differences were seen in terms of panicle number, panicle length and leaf width which were increased by 24.39, 13.46 and 13.33% in NT and 24.39, 10.10 and 6.67% in NH, respectively compared with those of NaCl treatment (Table 1A). Although the harvest index remained unchanged (Fig. 4B), 1000 seed weight and seed yield/plant were increased by ~ 30–34% (Fig. 4C) and ~ 25–27% (Fig. 4D) under NT and NH treatment, respectively compared to NaCl treatment. The major impact of TU and H_2_O_2_ alone treatments was seen in terms of panicle number and leaf length respectively which were increased by 14 and 18.3%, compared with those of WS control (Table 1A). In addition, the harvest index, 1000 seed weight and seed yield/plant were also increased by 13.19, 11.08 and 17.16% under TU and 15.87, 12.71 and 12.53% under H_2_O_2_ alone, respectively compared with those of WS control (Fig. 4A–C).

**Table 1:** Quantification of various growth [A] and PS-II related photosynthesis parameters [B] under different treatment conditions**.**

Treatments	Plant height (cm)	Leaf length	Leaf width	Panicle length(cm)	Panicle number	Chlorophyll (AU unit)
**A. Growth parameters**						
WS	107.7^**b**^ (± 0.6)	33.2^**c**^ (± 1.8)	1.6^**b**^ (± 0.08)	24.5^**a**^ (± 0.6)	5.0^**c**^ (± 0.2)	40.4^**b**^ (± 1.0)
TU	110.7^**a**^ (± 1.2)	35.8^**b**^ (± 1.6)	1.7^**b**^ (± 0.06)	24.7^**a**^ (± 0.4)	5.7^**a**^ (± 0.4)	45.1^**a**^ (± 1.1)
H_2_O_2_	110.6^**a**^ (± 1.2)	39.3^**a**^ (± 1.4)	1.8^**a**^ (± 0.04)	24.9^**a**^ (± 0.5)	5.6^**ab**^ (± 0.4)	40.4^**b**^ (± 1.3)
NaCl	95.9^**e**^ (± 0.5)	29.0^**d**^ (± 1.0)	1.5^**c**^ (± 0.04)	20.8^**c**^ (± 0.6)	4.1^**d**^ (± 0.3)	33.8^**c**^ (± .1.0)
NT	104.8^c^ (± 1.2)	32.6^c^ (± 1.4)	1.7^b^ (± 0.05)	23.6^b^ (± 0.5)	5.1^bc^ (± 0.3)	40.7^b^ (± 0.7)
NH	102.1^d^ (± 0.5)	31.8^c^ (± 0.6)	1.6^b^ (± 0.04)	22.9^b^ (± 0.2)	5.1^bc^ (± 0.2)	39.0^b^ (± 1.5)

Treatments	Photosynthetic rate (µMol CO_2_ m^-2^ s^-1^)	WUE	Fv/Fm	ETR	Quantum yield of PSII	NPQ (AU unit)
**B. Photosynthetic parameters**						
WS	6.2^**b**^ (± 0.7)	2.1^**a**^ (± 0.2)	0.720^**a**^ (± 0.03)	51.7^**ab**^ (± 1.8)	0.124^**b**^ (± 0.00)	2.1^**ab**^ (± 0.03)
TU	7.1^**ab**^ (± 0.5)	2.3^**a**^ (± 0.1)	0.734^**a**^ (± 0.01)	58.4^**a**^ (± 3.4)	0.144^**a**^ (± 0.01)	1.8^**b**^ (± 0.18)
H_2_O_2_	7.5^**a**^ (± 0.4)	2.4^**a**^ (± 0.7)	0.715^**a**^ (± 0.01)	60.5^**a**^ (± 6.5)	0.153^**a**^ (± 0.02)	2.4^**a**^ (± 0.53)
NaCl	1.9^**d**^ (± 0.5)	0.8^**c**^ (± 0.03)	0.614^**b**^ (± 0.04)	24.3^**d**^ (± 1.7)	0.087^**d**^ (± 0.003)	1.6^**b**^ (± 0.04)
NT	3.4^**c**^ (± 0.2)	1.3^**b**^ (± 0.1)	0.705^**a**^ (± 0.04)	48.9^**bc**^ (± 8.2)	0.136^**ab**^ (± 0.01)	1.8^**b**^ (± 0.02)
NH	3.9^**c**^ (± 1.1)	1.4^**b**^ (± 0.2)	0.717^**a**^ (± 0.01)	42.0^**c**^ (± 3.2)	0.102^**c**^ (± 0.01)	2.1^**ab**^ (± 0.49)

Total three foliar applications were given at vegetative, early anthesis and grain filling stages that corresponded to 40, 55 and 72 days post-transplantation, respectively. The quantitation was done after 5 days of 3rd foliar spray. The main flag leaf was taken for the analysis of photosynthetic parameters. Different treatment includes WS (DW + 0.01% Tween-20), TU (6.5 mM TU + 0.01% Tween-20) and H_2_O_2_ (10 mMH_2_O_2_ + 0.01% Tween-20), NaCl (22 g NaCl per pot). For NT and NH, a combined treatment of NaCl + TU and NaCl + H_2_O_2_ treatments were also given respectively. The values represent mean of ten biological replicate ± SE. Different letter indicates significantly different values (DMRT, *p* ≤ 0.05*).*

### Alteration of redox status and ionic distribution under TU and H_2_O_2_ supplementation

**Figure 5:**
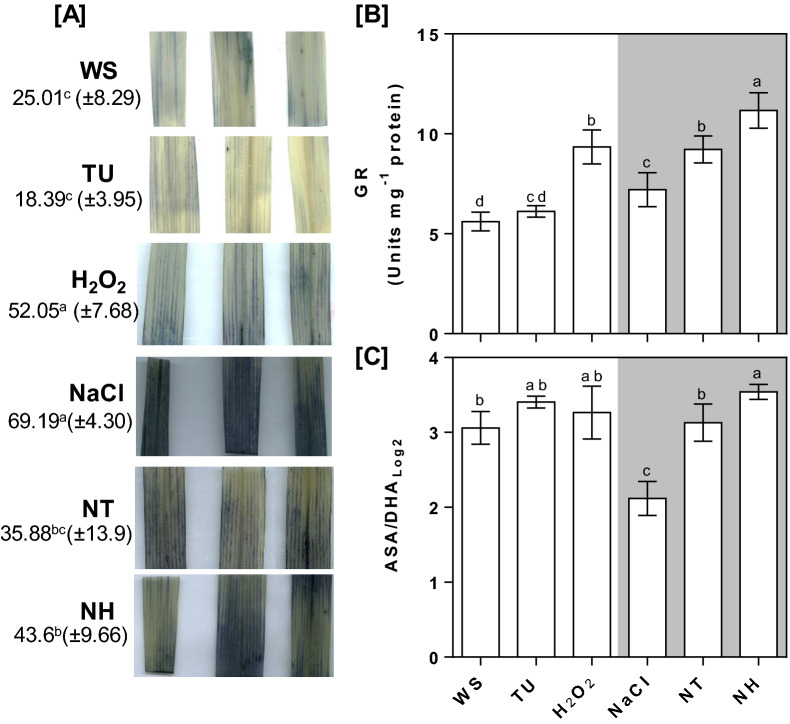
Measurement of the redox state in terms of the superoxide level, GR activity and ASA/DHA ratio. Pot-grown rice plants were given different treatments including WS (DW + 0.01% Tween-20), TU (6.5 mM TU + 0.01% Tween-20) and H_2_O_2_ (10 mM H_2_O_2_ + 0.01% Tween-20), NaCl (22 g NaCl per pot). NT and NH denote combined treatment of NaCl + TU and NaCl + H_2_O_2_ treatments, respectively. A total of three foliar applications were given at vegetative, early anthesis and grain filling stages that corresponded to 40, 55 and 72 days post-transplantation, respectively. At 5th day after 3rd foliar spray, flag leaves were harvested for NBT based histochemical staining (**A**), quantification of GR activity (**B**) and ascorbate/dehydroascorbate (ASA/DHA) ratio (**C**). NBT stain intensity was quantified using image J software (version 1.53d; https://imagej.nih.gov) and represented as average arbitrary units (± S.D.). All the values are mean of triplicates ± SD. Different letters indicate significantly different values (DMRT, *p* ≤ 0.05*)*.

Histochemical staining revealed increased accumulation of O_2_^.-^ radical, specifically under NaCl-treated flag leaf. No significant change in NBT stain intensity was observed for TU, NT and NH treatments. On the other hand, a moderate increase in NBT staining was observed under H_2_O_2_ alone treatments (Fig. 5A). The activity of GR and ASA/DHA ratio were increased and decreased by 28.5 and 30.7%, in NaCl-treated seedlings compared with those of control. Both these parameters were further increased by 27.94 and 47.6% (NT) and 55 and 67% (NH), respectively compared with that of NaCl treatment (Fig. 5B,C). Although GR activity remained unchanged in TU, it was increased by 66.6% under H_2_O_2_ alone treatment (Fig. 5B). No significant change was observed for the ASA/DHA ratio under TU and H_2_O_2_ alone treatment, compared to control (Fig. 5C).

Under NaCl stress, maximum Na^+^ accumulation was observed in young leaves (YL), where it increased by 403.8%, followed by old leaves (OL) and developing inflorescences (DI), where it increased by 159.94 and 70.21%, respectively, compared with WS-treated plants (Supplementary table 3A). In contrast, under NT treatment, Na^+^ accumulation from YL and DI organs wasecreased by 21.48 and 53.75%, respectively; however, in OL, it was increased by 15.97%, compared with those of NaCl-treated plants. In NH-treated plants, the Na^+^ accumulation was reduced in all the three tested organs including YL, OL and DI by 38.57, 21.48 and 50%, respectively compared with those of NaCl-treated plants. No significant change in Na^+^ accumulation was observed in TU while H_2_O_2_ alone treatments resulted in 34.18% decrease and 45.91% increase the Na^+^ accumulation in the YL and OL respectively compared to those of WS control (Supplementary table 3A). The increased Na^+^ levels in NaCl-treated plants was concomitant with reduced K^+^ accumulation by 50.43, 63.98 and 24.09% in YL, OL and DI organs, respectively compared with those of WS- treated control. The extent of K^+^ reduction was restored in all the three organs under NT and NH treatments. However, the maximal reversal of 105.34 and 112.77%, under NT and NH treatments, respectively was observed in YL organ compared with those of NaCl treatment. Enhanced K^+^ accumulation was also observed under TU and H_2_O_2_ alone treatments compared with WS controls, with the exception of OL in TU-treated plants, where K^+^ levels remained unchanged (Supplementary table 3B).

### Photosynthetic responses under TU and H_2_O_2_ supplementation

The overall process of photosynthesis was negatively affected in NaCl-treated plants. The major impact was seen in terms of photosynthetic rate (PR), water use efficiency (WUE) and electron transport rate (ETR) which were decreased by 69.35, 61.90 and 53%, respectively compared with those of WS control. In contrast, significant photosynthetic recovery was noticed under both NT and NH treatments as these parameters were increased by 78.95, 62.5 and 101.23% in NT and 105.26, 75 and 72.84% in NH compared with that of NaCl treatment. In addition, quantum yield of photosystem II (PS-II yield) and non photochemical quenching (NPQ) were also increased specifically under NT (56.32%) and NH (31.25%), respectively compared with those of NaCl treatment (Table 1B). The photosynthesis and PS-II yield were also affected under TU and H_2_O_2_ alone treatments as indicated by 14.52 and 20.97% increase in TU and 16.13 and 23.39% increase in H_2_O_2_ treatment compared with those of WS control (Table 1B).

### Modulation of source-sink homeostasis under TU and H_2_O_2_ supplementation

**Figure 6:**
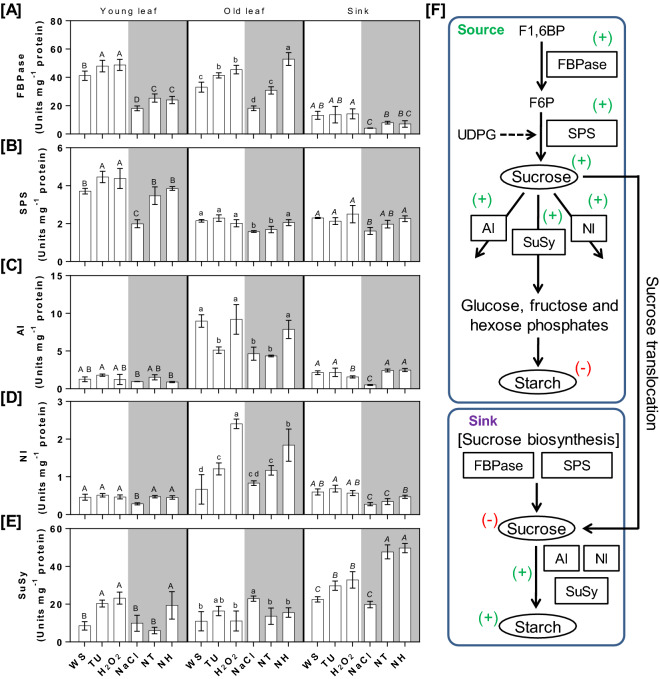
Quantification of enzymes and metabolites regulating source-sink equilibrium. Pot-grown rice plants were given different treatments including WS (DW + 0.01% Tween-20), TU (6.5 mM TU + 0.01% Tween-20) and H_2_O_2_ (10 mM H_2_O_2_ + 0.01% Tween-20), NaCl (22 g NaCl per pot). NT and NH denote combined treatment of NaCl + TU and NaCl + H_2_O_2_ treatments, respectively. A total of three foliar applications were given at vegetative, early anthesis and grain filling stages that corresponded to 40, 55 and 72 days post-transplantation, respectively. At 5th day after 3rd foliar spray, flag leaves were harvested and activities of various enzymes including FBPase (Fructose 1,6 bis-phosphatase; **A**); SPS (Sucrose phosphate synthetase; **B**); AI (Acid soluble invertase; **C**); NI (Neutral invertase; **D**) and SuSy (Sucrose synthase; **E**) were measured. All values represent mean of triplicates ± SD. Different letters indicate significantly different values (DMRT, *p* ≤ 0.05*)*. The right-hand panel represent the overview of sucrose biosynthesis/breakdown pathway operated in source and sink organs (**F**). The rectangles and ovals represent the enzymes and metabolites, respectively, quantified in the present study. The positive (+) and negative (−) labels denotes the role of a particular enzymes/metabolites for increasing and decreasing source/sink strength, respectively.

Akin to photosynthesis, the activities of key enzymes determining source (SPS and FBPase) and sink (AI, NI and SuSy) strength were also negatively impacted in NaCl-treated plants; except, for SuSy which was increased by 108.75%, in old leaves compared with WS control. The ameliorative effects were observed under both NT and NH treatments, at the level of source as well as sink. Besides, differential nature of NT and NH was also seen, especially in the source leaves. For instance, the SPS activity was increased by 93.47, 29.75 and 40.37% in NH-treated YL, ML and DI organs, respectively compared with those of NaCl treatment. However, under NT treatment, increased SPS activity was limited to YL (74.7%) and DI (22.36%) organ only. Unlike SPS, FBPase activity was significantly increased under both NT and NH, in all the three tested organs, compared with those of NaCl treatment. The sucrose degradation pathway was also activated under NT and NH treatment conditions. In source organs like YL and OL, the NI activity was increased 66.43 and 40.44% in NT and 59.05 and 120.97% in NH, respectively compared with those of NaCl treatment (Fig. 6C). Besides, in YL organ, NI and SuSy activities were also increased by 59.21 and 96.05% in NT and NH, respectively compared with those of NaCl treatment (Fig. 6D,E). In sink (DI), the activities of AI and SuSy were increased respectively by 381.99 and 141.38% in NT and 391.74 and 151.54% in NH compared with those of NaCl treatment (Fig. 6D,E). Under TU and H_2_O_2_ alone treatments, well-coordinated sucrose biosynthesis, mediated by SPS and FBPase, was observed in YL (Fig. 6A,B). In parallel, SuSy mediated sucrose breakdown was also observed under YL and DI; while AI was found to be activated in OL organ (Fig. 6C–E).

The sucrose and starch levels were differentially affected under NaCl with/without TU or H_2_O_2_ supplementation. In source organs, the sucrose level under NaCl stress was either reduced by 27.65% in YL or remained unchanged in OL compared with those of WS control. In contrast, the sucrose level was significantly increased, in both YL and OL organs, by 46.27 and 25.34% in NT and 58.71 and 30.35% in NH, respectively compared to that of NaCl-treated plants. Unlike source, sucrose was reduced by 32.32% in NaCl-treated DI compared to WS control. Under NT and NH treated DI organ, the sucrose level was decreased by 19.08 and 40.46%, respectively compared with those of NaCl treatment (Supplementary table 4A). Compared to WS control, starch level in NaCl-treated source leaves was increased by 53.42 (YL) and 48.48% (OL); while it was decreased by 48.48% in DI organ. In contrast, under both NT and NH treatments, the starch level was found to be significantly decreased and increased in source (YL and OL) and sink (DI) organs, respectively (Supplementary table 4B).

### Understanding treatment-variable interactions through PCA based clustering

**Figure 7:**
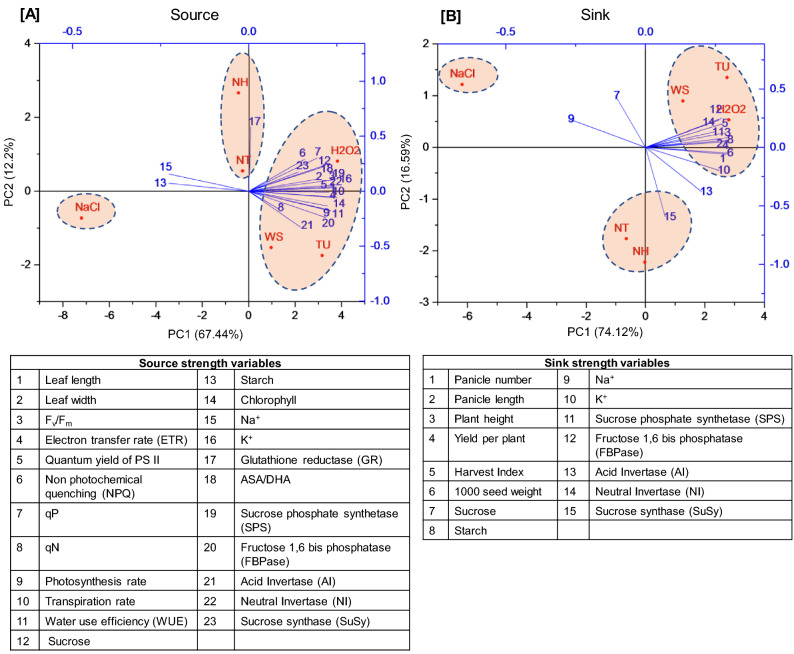
Principal component analysis on field grown plants to understand treatment-variable interaction. The principal component analysis (PCA) was performed to identify the variables associated with different treatments including WS (DW + 0.01% Tween-20), TU (6.5 mM TU + 0.01% Tween-20) and H_2_O_2_ (10 mM H_2_O_2_ + 0.01% Tween-20), NaCl (22 g NaCl per pot). NT and NH denote combined treatment of NaCl + TU and NaCl + H_2_O_2_ treatments, respectively. The biplots were generated independently for young leaf (source; **A**) and developing inflorescence (sink; **B**) using the variables responsible for mediating source and sink strength, respectively. The lines originating from central point of biplots indicate positive or negative correlations of different variables; where their closeness indicates correlation strength with particular treatment. The variables were represented in the form of numeric values with its details in associated tables.

PCA was performed on the entire data sets to identify the key variables associated under various treatment conditions. In source leaves, different treatments were grouped into three categories (Fig. 7A). The first category contained photosynthesis and sucrose biosynthesis and breakdown related parameters and was found to be associated with WS and TU and H_2_O_2_ treatments. The second category had only one attribute (GR activity) which was associated with NT as well as NH treatments. The third category represented NaCl stress and this was not associated with any of the variables (Fig. 7A). Similarly, three major categories were also identified in sink organs. The first category included plant growth and yield related parameters which was associated with WS, TU and H_2_O_2_ treatments. The second category included source-sink homeostasis related enzymes/metabolites, associated with NT and NH treatments. The third category of sucrose and Na^+^ content was associated with NaCl treatment (Fig. 7B).

## Discussion

Redox homeostasis is an essential constituent for sustained maintenance of plant growth and survival under stress conditions. The present study has evaluated widely used redox modulators like TU and H_2_O_2_ for mitigating salt stress in rice. In our previous studies, we have successfully demonstrated TU-mediated responses at multiple levels of organization. At the physiological level, TU improved source-to-sink relationship leading to increased crop yield^34^ while at the molecular level, it improved cellular energetics^35^, co-ordinated calcium and abscisic acid (ABA) signaling events^36^, maintained plant-water homeostasis^37^, enhanced antioxidant defense^20^ and improved sulphur metabolism^23^. Additionally, TU effectiveness has also been demonstrated under other types of abiotic stresses^38^, like drought, heat, UV radiation, metal stress. Similarly, H_2_O_2_ is another broad range effective PBR whose ameliorative potential has been demonstrated against various abiotic stresses^39^.

Considering their chemical nature, TU (a ROS scavenger) and H_2_O_2_ (a biological ROS) are expected to have a contrasting effect on the cellular redox state, which is described as an integrated ratio of reduced to oxidized form of all the redox couples present inside the cell. At whole plant level, the redox state is regulated by ROS-scavenging/producing enzymes and antioxidant metabolites^40^. Initially, the post-germination phenotyping was performed at seedling stage with variable doses of NaCl (ranging from 50 to 100 mM) and 50 mM was identified as the IC50 dose at which plant biomass is reduced by 48.15% compared with those of control (Supplementary Fig. 1). Similar dose-dependent analysis was also performed with TU and H_2_O_2_ (ranging from 1 to 100 μM). On the basis of biomass, 7.5 and 1.0 μM were identified as optimum doses of TU and H_2_O_2_ which were selected for all the medium supplementation studies (Supplementary Fig. 2). In spite of having a contrasting chemistry, *in planta* supplementation of both TU as well as H_2_O_2_ increased the redox capacity and improved the plant growth potential under NaCl stress conditions (Fig. 1). Such an overlapping response clearly indicated that H_2_O_2_ supplementation also generates reducing redox environment of the plants, which is shown by the significant increase in activities of antioxidant enzymes like SOD, CAT and GR under both control and NaCl stress conditions (Fig. 2A–C). The ability of H_2_O_2_ to trigger the plant’s antioxidant defense has also been demonstrated in various crops like soybean^41^, rice^42^ and wheat^43^. Unlike H_2_O_2_, the enzymatic antioxidants were less pronounced in TU-treated control plants, indicating that non-enzymatic antioxidants were involved in maintaining reducing redox environment. Earlier, TU-mediated activation of non-enzymatic antioxidants has been demonstrated in *Brassica juncea*^44^. Since most of the NaCl-induced damages including biomass reduction as well as decreased root and shoot length were associated with ROS-induced redox imbalance^45^, therefore, the NaCl ameliorative potential of TU and H_2_O_2_ could largely be associated with their ability to generate reducing redox environment inside the plants.

In addition to redox balance, improved K^+^ retention was also observed under both NT- and NH- treated plants (Fig. 2F; Supplementary table 2), which might have supported the improved plant phenotype under NaCl stress conditions (Fig. 1). A positive correlation between salt tolerance and higher K^+^ accumulation has been demonstrated in crops like rice and barley^46^. The NaCl-induced K^+^-leakage is associated with ROS production which activates K^+^-efflux channels including guard cell outward rectifying K^+^ channel (GORK) and stelar K^+^ outward rectifier (SKOR)^47^. The higher K^+^ retention ability of TU and H_2_O_2_ further substantiates our hypothesis that both these modulators have an overlapping capacity to generate reducing redox environment. This was also supported by TU and H_2_O_2_ dependent upregulated expression of *HAK21* (Fig. 3A), which is known to maintain K^+^ ion homeostasis under NaCl stress conditions^8^. Other NaCl stress responsive genes, especially *LEA*^1^*, dehydrin*^7^*, TSPO*^10^ *and EN20*^11^, known for imparting NaCl tolerance in redox dependent manner, were also upregulated suggesting their association in the tolerant phenotype observed under NT and NH treatments. Incidentally, their redox-dependent regulation has already been demonstrated^48–50^, further justifying their regulation through TU and H_2_O_2_ dependent manner. In addition, the expression of most of the tested NaCl tolerance related genes were also increased constitutively even under stress-free conditions (Fig. 3). This result indicated that during TU/H_2_O_2_ pre-treatment phase, the seedlings were better equipped to face the ensuing NaCl stress exposure.

Similar to nutrient medium supplementation, foliar-applied TU and H_2_O_2_ was effective in maintaining the ROS and redox homeostasis as evident by decreased superoxide load under NT and NH treatments compared to NaCl (Fig. 5). Further H_2_O_2_ mediated increase in NBT staining under control conditions (Fig. 5A) may be considered as pro-oxidant behavior by imposing mild oxidative stress. Foliar-application of TU and H_2_O_2_ was also found effective in enhancing plant growth and yield under field conditions in both control and NaCl stress conditions (Fig. 4), substantiating their agronomic feasibility. Considering the difference in plant size and possible degradation under the natural sunlight, we have selected higher doses of TU (6.5 mM) and H_2_O_2_ (1 mM) for foliar supplementation, which were also used previously in various other crops^19^. The significant reduction in PS-II efficiency under NaCl treatment was reflected by a drastic reduction in plant growth as well as yield (Fig. 4; Table 1). The redox balancing, better K^+^ retention and improved photosynthesis were identified as key attributes responsible for mediating TU and H_2_O_2_ dependent amelioration under NaCl stress conditions. Of these, K^+^ retention and dynamic ROS production in leaf mesophyll cell have been shown to be well-correlated with salt tolerance in rice at reproductive stage, in both greenhouse as well as field conditions^51^. Besides, K^+^ and ROS are considered as major signaling regulators and hence, have potential capacity to alter plant growth^52,53^. In line with K^+^ retention, we also observed reduced Na^+^ accumulation under both NT and NH treatments, especially in YL and DI which represent the main organs for photosynthesis and reproduction, respectively (Supplementary table 3).

The Na^+^ dependent inhibition of photosynthesis has been reported in various crops like rice^54^, barley^55^ and wheat^56^. The lower Na^+^ accumulation as observed in our study could have facilitated the improved photosynthesis (Table 1B) and overall plant growth and vigor. Although, there are multiple ways by which Na^+^ imposes toxicity; imbalance in Na^+^/K^+^ ratio is a major contributor of Na^+^ mediated reduction in plant photosynthesis^57^. Most of the PS-II efficiency components (photosynthesis rate, WUE, Fv/Fm, ETR and quantum yield of PSII) were equally improved; however, NPQ was identified as NH-specific parameter (Table 1B). The excess energy dissipation through NPQ protects the photosystem from ROS mediated photoinhibition. This is supported by H_2_O_2_ dependent regulation of xanthophylls^58^, which represent the major components for maintaining higher NPQ and low ROS under salt stress conditions^59^.

In plants, sucrose and starch are considered as key indicators of source (leaves) and sink (developing inflorescence) strengths, respectively^60^. The higher and lower levels of sucrose and starch in source and vice versa in sink, clearly indicated a synchronized source-sink relationship in both NT- and NH- treated plants (Supplementary Table 4). Most of the Calvin cycle enzymes are regulated in redox dependent manner^61^. For example, FBPase (a sucrose biosynthesis enzyme) is active under the reducing redox environment^62^. Thus, owing to the generation of reducing redox environment (Fig. 5C), the overall source strength was high resulting in the improved growth potential of NT- and NH- treated plants. This was also supported by PCA in source leaf, wherein NT- and NH- treatments were found to be associated with GR activity (Fig. 7A). GR is the major enzyme for regenerating GSH and hence, is responsible for maintaining reducing redox conditions in the plant^63^. Similarly, overexpression of SPS (a rate-limiting enzyme of sucrose biosynthesis^64^ has been achieved in sugarcane transgenic lines which accumulated more sucrose with significant increase in plant height and stalk number compared to non-transgenic control^65^. In addition, de-regulation between FBPase and SPS activities was also observed, suggesting the onset of senescence in NT-treated OL organ. This is in contrast with NH wherein, increased AI and NI activities avoided the feedback inhibition and hence, sucrose biosynthesis was found to be active in both YL and OL organs (Fig. 6A,B). In both NT- and NH- treated sink organs, SuSy and AI were preferred (over NI) for breaking down the sucrose metabolites (Fig. 6C–E). The higher starch content will not only improve sink strength but also, the overall fitness of the plant^66^. In contrast, NI activity was also observed in OL organ, which can provide hexoses, as they are necessary for restricting ROS level^67^ and also to fulfill energy demand of plants under stress conditions^68^. Similar to our results, *SuSy* overexpression^69^ or mutation in *AI*^70^ have been shown to increase or decrease the grain weight, respectively, in transgenic lines of rice. Taken together, the approach of using TU and H_2_O_2_ can be seen as an alternative to the genetic methods of enhancing source-sink strength in plants under NaCl stress conditions.

A significant enhancement in growth and yield was also noticed under TU and H_2_O_2_ alone treatments (Fig. 4; Table 1). Although the absolute levels of sucrose and starch remained unchanged (Supplementary table 4), the higher activities of SPS and FBPase in YL and SuSy in both YL and DI organs clearly indicated that source-sink strength was boosted in TU and H_2_O_2_ treated plants (Fig. 6). Further, PCA also indicated an overlapping response as most of the growth and yield related attributes were grouped together with between TU and H_2_O_2_ alone treatments (Fig. 7). Thus, the positive impact of TU and H_2_O_2_ under both control and NaCl stress conditions greatly increases their versatility to be applied under the realistic field scenario.

## Conclusions

In conclusion, the study highlights that despite having contrasting redox chemistry, both TU and H_2_O_2_ impart comparable level of NaCl stress tolerance in rice to a comparable extent. Both TU and H_2_O_2_ upregulated the expression of NaCl stress responsive genes in a constitutive manner, without showing any significant growth reduction under control conditions. In addition, reducing redox status was maintained along with better K^+^ retention and upregulated expression of NaCl stress tolerant genes like *HAK21*, *LEA1, dehydrin, TSPO* and *EN10* under both NT and NH treated seedlings, resulting in improved growth. Under both control and NaCl stress conditions, foliar-supplemented TU and H_2_O_2_ improved the growth and yield attributes of the plants. The ameliorated phenotype under NT and NH was associated with reduced Na^+^ accumulation, improved photosynthesis efficiency and better source-sink relationship. Taken together, the results of this study suggest that the maintenance of reduced redox status acts as a “central” regulator for mitigating NaCl-induced damages. Besides, it also extends the concept of using redox modulators for improving rice crop productivity under the realistic field scenario.

## Supplementary Information

Supplementary Information 1.

Supplementary Information 2.
