# Supplementary material for: Thiourea and hydrogen peroxide priming improved K+ retention and source-sink relationship for mitigating salt stress in rice
Source: Sci Rep. 2021 Feb 4;11:3000. doi: 10.1038/s41598-020-80419-6 (PMC7862675; doi:10.1038/s41598-020-80419-6)
Supplement: Supplementary file 1 — Supplementary Information 1. [file 41598_2020_80419_MOESM1_ESM.doc]

**Thiourea and hydrogen peroxide priming improved K^+^ retention and source-sink relationship for mitigating salt stress in rice**

Pandey M^1,2^, Radha Krishna P^1^, Srivastava AK^1,2^*, Suprasanna P^1,2^*

*^1^Nuclear Agriculture and Biotechnology Division, Bhabha Atomic Research Centre, Mumbai 400085, India; ^2^Homi Bhabha National Institute, Mumbai-400095, India*

***For correspondence:*** *Penna Suprasanna (*[*penna888@yahoo.com*](mailto:penna888@yahoo.com) *); Ashish Kumar Srivastava (ashishbarc@gmail.com)*

**Running title: Thiourea and hydrogen peroxide mitigate salt stress in rice**

**Supplementary Table-1: Details of the primers used for gene-specific quantitative real-time PCR.**

| **Gene details** | **Primer sequence** |
| --- | --- |
| Trehalose–phosphate phosphatase  (TPP; LOC_Os02g44235) | For- TGCTGAAAAGGTTACCAAGTCC |
|  | Rev- TGCGACCAGTTTCCAATCCT |
| Late embryogenic abundant protein group -3  (LEA3; LOC_Os04g52110) | For- CCATGCTGCTCGGTGGC |
|  | Rev- GTACGACGGCCTCGGATGAT |
| Phosphatidylinositol-4-phosphate 5-kinase  (PIPK; LOC_Os04g59540) | For- GCAATCTGGAACGACACAGC |
|  | Rev- TCCACTCCCACCAGTAACGA |
| Translocator protein (TSPO; LOC_Os05g05930) | For- GAGCCGGGATCCGAGGAA |
|  | Rev- TAGAACGAGGCGGCCATCAG |
| Early nodulin 20  (EN20; LOC_Os06g46740) | For- TCGCCGTCGGTTTCACCTA |
|  | Rev- GTTCGCCCCATCGGTCC |
| Potassium transporter  (HAK21; LOC_Os03g37930) | For- CTCACTGGGACGAACAGGAT |
|  | Rev- CCCAATGCCGTGCTCAAAAG |
| Dehydrin  (DHD; LOC_Os11g26760) | For- GCAGATGATGGGGAACACCG |
|  | Rev- TCCATGAAGCCCTTCTTCTCG |
| Late embryogenic abundant protein group-1  (LEA1; LOC_Os03g20680) | For- AGGCGACCAAGAACAAGCTG |
|  | Rev- TTGAACTCCGTCGCCTTCTG |
| Tubulin  (Tub; LOC_Os01g59150.1) | For- TTTGTGTTCGGGCAATCTGGTG |
|  | Rev- AGTGGCATACTTGGAATCCTTGC |

**Supplementary Table-2: Time dependent accumulation of Na^+^, K^+^ and Na^+^/K^+^ ratio in the leaf of rice seedling subjected to different treatments.** Hydroponically grown seedlings (14 d) were subjected to different treatments including control (Yoshida medium), NaCl (50 mM), TU (7.5 µM), NaCl (50 mM) + TU (7.5 µM) [NT], H_2_O_2_ (1 µM) and NaCl (50 mM) + H_2_O_2_ (1 µM) [NH]. For NT and NH, 24 h pretreatment of 7.5 µM TU and 1 µM H_2_O_2_ respectively was also given. After 1, 6, 24 and 48 h of treatment the leaf tissue was harvested and analyzed for activities of SOD (A), CAT (B), GR (C) and APX (D) and levels of Na^+^ (E) and K^+^ (F). All the values are mean of triplicates ± SD. Different letters indicate significantly different values (DMRT, *p* ≤ 0.05*)*.

| **Treatments** | **1 h** | **6 h** | **24 h** | **48 h** |
| --- | --- | --- | --- | --- |
| **A. SOD (Units mg^-1^ protein)**  **90** | | | | |
| **Control** | 3.39**^e^** (± 0.15) | 4.94**^b^** (± 0.55) | 4.62**^b^** (± 0.52) | 4.37**^c^** (± 0.15) |
| **TU** | 4.22**^d^** (± 0.09) | 4.71**^b^** (± 0.17) | 4.48**^b^** (± 0.66) | 3.78**^c^** (± 0.45) |
| **H_2_O_2_** | 4.53**^d^** (± 0.05) | 5.95**^ab^** (± 0.10) | 4.62**^b^** (± 0.38) | 5.57**^b^** (± 0.15) |
| **NaCl** | 5.51**^c^** (± 0.69) | 6.28**^a^** (± 0.46) | 5.04**^b^** (± 0.36) | 6.79**^a^** (± 0.62) |
| **NT** | 7.14^a^ (± 0.47) | 6.71**^a^** (± 0.46) | 6.33**^a^** (± 0.70) | 6.27^a^**^b^** (± 0.47) |
| **NH** | 6.35**^b^** (± 0.36) | 7.07**^a^** (± 1.50) | 6.53**^a^** (± 0.82) | 6.08^a^**^b^** (± 0.70) |
| **B. CAT (Units mg^-1^ protein)** | | | | |
| **Control** | 3.31**^c^** (± 0.40) | 3.52^c^ (± 0.27) | 3.25^c^ (± 0.30) | 4.36^c^ (± 0.70) |
| **TU** | 3.33^c^ (± 0.51) | 2.52^d^ (± 0.99) | 5.17^b^ (± 0.90) | 4.97^bc^ (± 1.00) |
| **H_2_O_2_** | 4.96^b^ (± 0.70) | 5.65^b^ (± 0.50) | 5.04^b^ (± 0.30) | 5.43^bc^ (± 0.90) |
| **NaCl** | 5.48^b^ (± 0.78) | 5.12^b^ (± 0.18) | 7.34^a^ (± 0.41) | 7.24^a^ (± 0.44) |
| **NT** | 4.64^b^ (± 0.25) | 4.77^b^ (± (0.56) | 5.06^b^ (± 0.63) | 5.58^bc^ (± 0.49) |
| **NH** | 7.60^a^ (± 0.37) | 6.87^a^ (± 0.21) | 5.53^b^ (± 0.73) | 5.94^b^ (± 0.48) |
| **C. GR (Units mg^-1^ protein)** | | | | |
| **Control** | 4.82^d^ (±0.45) | 5.18^c^ (±0.33) | 6.09^c^ (±0.80) | 6.29^c^ (±0.58) |
| **TU** | 6.84^c^ (±0.39) | 6.82^b^ (±0.82) | 7.16^bc^ (±0.04) | 7.00^c^ (±0.64) |
| **H_2_O_2_** | 7.61^b^ (±0.66) | 7.20^b^ (±0.57) | 8.35^b^ (±0.51) | 8.96^b^ (±0.20) |
| **NaCl** | 6.42^c^ (±0.21) | 7.08^b^ (±0.39) | 7.63^b^ (±0.84) | 8.86^b^ (±0.72) |
| **NT** | 9.00^a^ (±0.45) | 9.51^a^ (±0.48) | 10.37^a^ (±0.68) | 12.32^a^ (±0.56) |
| **NH** | 9.24^a^ (±0.01) | 9.36^a^ (±0.41) | 10.19^a^ (±1.39) | 11.64^a^ (±1.28) |
| **D. APX (Units mg^-1^ protein)** | | | | |
| **Control** | 1.72^e^ (±0.01) | 1.9^b^ (±0.05) | 1.59^a^ (±0.07) | 1.38^bc^ (±0.26) |
| **TU** | 1.78^e^ (±0.01) | 1.4^c^ (±0.05) | 0.23^c^ (±0.21) | 1.65^b^ (±0.21) |
| **H_2_O_2_** | 2.36^c^ (±0.23) | 1.54^c^ (±0.06) | 0.48^bc^ (±0.35) | 1.28^bc^ (±0.30) |
| **NaCl** | 2.94^b^ (±0.10) | 1.39^c^ (±0.19) | 0.43^bc^ (±0.09) | 0.96^c^ (±0.09) |
| **NT** | 3.5^a^ (±0.16) | 2.94^a^ (±0.16) | 1.04^ab^ (±0.59) | 1.32^bc^ (±0.16) |
| **NH** | 2.09^d^ (±0.34) | 0.75^d^ (±0.34) | 0.79^bc^ (±0.42) | 2.11^a^ (±0.24) |
| **E. Na^+^ content (% DW)** | | | | |
| **Control** | 0.220**^c^** (± 0.01) | 0.215**^d^** (± 0.01) | 0.260**^de^** (± 0.02) | 0.220**^d^** (± 0.01) |
| **TU** | 0.324**^b^** (± 0.03) | 0.308**^c^** (± 0.03) | 0.286**^d^** (± 0.02) | 0.296**^d^** (± 0.02) |
| **H_2_O_2_** | 0.223**^c^** (± 0.02) | 0.225**^d^** (± 0.02) | 0.233**^e^** (± 0.02) | 0.226**^d^** (± 0.01) |
| **NaCl** | 0.388**^a^** (± 0.01) | 0.482**^a^** (± 0.01) | 0.773**^a^** (± 0.02) | 2.730**^a^** (± 0.04) |
| **NT** | 0.355**^a^** (± 0.03) | 0.446**^b^** (± 0.01) | 0.705**^b^** (± 0.01) | 2.477**^b^** (± 0.08) |
| **NH** | 0.251**^c^** (± 0.02) | 0.325**^c^** (± 0.01) | 0.639**^c^** (± 0.03) | 2.086**^c^** (± 0.05) |
| **F. K ^+^ content (% DW)** | | | | |
| **Control** | 2.287**^bc^** (± 0.24) | 2.146**^b^** (± 0.18) | 2.290**^b^** (± 0.30) | 2.151**^b^** (± 0.14) |
| **TU** | 2.660**^abc^** (± 0.50) | 3.166**^a^** (± 0.31) | 2.750**^a^** (± 0.26) | 2.870**^a^** (± 0.23) |
| **H_2_O_2_** | 2.973**^a^** (± 0.34) | 2.683**^a^** (± 0.25) | 2.543**^ab^** (± 0.18) | 2.981**^a^** (± 0.51) |
| **NaCl** | 2.104**^c^** (± 0.15) | 1.903**^b^** (± 0.05) | 1.271**^c^** (± 0.06) | 0.877**^c^** (± 0.04) |
| **NT** | 2.917**^a^** (± 0.22) | 2.883**^a^** (± 0.21) | 2.581**^ab^** (± 0.19) | 2.169**^b^** (± 0.18) |
| **NH** | 2.742**^ab^** (± 0.31) | 3.010**^a^** (± 0.52) | 2.212**^bc^** (± 0.21) | 1.802**^b^** (± 0.09) |

**Supplementary Table-3: The differential accumulation of the Na^+^ and K^+^ accumulations in young leaf, old leaf and developing sink.** Total three foliar applications were given at vegetative, early anthesis and grain filling stages that corresponded to 40, 55 and 72 d post-transplantation, respectively. Specified tissues from different treatment were harvested after 5 d of 3^rd^ foliar spray. Different treatment includes WS (DW + 0.01% Tween-20), TU (6.5 mM TU+ 0.01% Tween-20) and H_2_O_2_ (10 mM H_2_O_2_ + 0.01% Tween-20), NaCl (22 g NaCl per pot). NT and NH denote combined treatment of NaCl+TU and NaCl+H_2_O_2_ treatments, respectively. All the values are mean of triplicates ± SD. Different letters indicate significantly different values (DMRT, *p* ≤ 0.05*)*.

| **A. Na^+^ (% DW)** | | | |
| --- | --- | --- | --- |
| **Treatments** | **Young leaf** | **Old leaf** | **Developing sink** |
| **WS** | 0.158**^d^** (± 0.03) | 0.342**^e^** (± 0.04) | 0.47**^b^** (±0.02) |
| **TU** | 0.156**^d^** (± 0.04) | 0.364**^e^** (± 0.06) | 0.42**^bc^** (±0.05) |
| **H_2_O_2_** | 0.104**^d^** (± 0.01) | 0.499**^d^** (± 0.01) | 0.49**^b^** (±0.03) |
| **NaCl** | 0.796**^a^** (± 0.04) | 0.889**^b^** (± 0.05) | 0.80**^a^** (±0.02) |
| **NT** | 0.625**^b^** (± 0.02) | 1.031**^a^** (± 0.07) | 0.37**^c^** (±0.02) |
| **NH** | 0.489**^c^** (± 0.04) | 0.698**^c^** (± 0.03) | 0.40**^c^** (±0.01) |
| **B. K ^+^ (% DW)** | | | |
| **WS** | 3.175**^b^** (± 0.58) | 4.014**^b^** (± 0.24) | 1.694**^c^** (±0.04) |
| **TU** | 4.089**^a^** (± 0.20) | 3.842**^b^** (± 0.11) | 2.062**^a^** (±0.09) |
| **H_2_O_2_** | 3.690**^ab^** (± 0.16) | 5.674**^a^** (± 0.16) | 2.196**^a^** (±0.09) |
| **NaCl** | 1.574**^c^** (± 0.12) | 1.446**^e^** (± 0.32) | 1.286**^d^** (±0.04) |
| **NT** | 3.232**^b^** (± 0.29) | 2.078**^d^** (± 0.18) | 1.946**^b^** (±0.23) |
| **NH** | 3.349**^b^** (± 0.22) | 2.938**^c^** (± 0.35) | 1.972**^b^** (±0.22) |

**Supplementary Table-4: Differential sucrose and starch content in young leaf, old leaf and developing sink under different treatment conditions.** Total three foliar applications were given at vegetative, early anthesis and grain filling stages that corresponded to 40, 55 and 72 d post-transplantation, respectively. Specified tissues from different treatment were harvested after 5 d of 3^rd^ foliar spray. Different treatment includes WS (DW + 0.01% Tween-20), TU (6.5 mM TU+ 0.01% Tween-20) and H_2_O_2_ (10 mM H_2_O_2_ + 0.01% Tween-20), NaCl (22 g NaCl per pot). NT and NH denote combined treatment of NaCl+TU and NaCl+H_2_O_2_ treatments, respectively. All the values are mean of triplicates ± SD. Different letters indicate significantly different values (DMRT, *p* ≤ 0.05*)*.

| **A. Sucrose (mg g^-1^ DW)** | | | |
| --- | --- | --- | --- |
| **Treatments** | **Young leaf** | **Old leaf** | **Developing sink** |
| **WS** | 62.93**^ab^** (± 7.80) | 43.07**^c^** (± 8.40) | 19.80**^b^** (± 1.87) |
| **TU** | 69.93**^a^** (± 8.80) | 39.80**^c^** (± 3.47) | 26.00**^a^** (± 2.93) |
| **H_2_O_2_** | 73.47^a^ (±14.67) | 66.53**^a^** (± 5.40) | 19.27**^b^** (± 2.20) |
| **NaCl** | 45.53**^b^** (± 5.87) | 43.93**^c^** (± 3.07) | 26.20**^a^** (± 2.40) |
| **NT** | 66.60**^a^** (± 13.00) | 55.07**^b^** (± 3.20) | 21.20**^ab^** (± 3.13) |
| **NH** | 72.27**^a^** (± 6.33) | 57.27**^b^** (± 4.33) | 15.60**^b^** (± 4.80) |
| **B. Starch (mg g^-1^ DW)** | | | |
| **WS** | 10.73**^b^** (± 0.8) | 13.20**^b^** (± 1.07) | 44.00**^ab^** (± 1.93) |
| **TU** | 9.87**^b^** (± 0.53) | 11.67**^bc^** (± 0.40) | 48.60**^a^** (± 6.07) |
| **H_2_O_2_** | 11.93**^b^** (± 1.07) | 9.93**^c^** (± 0.80) | 48.80**^a^** (± 2.73) |
| **NaCl** | 16.47**^a^** (± 1.06) | 19.60**^a^** (± 3.33) | 22.67**^c^** (± 5.20) |
| **NT** | 11.53**^b^** (± 2.13) | 13.00**^b^** (± 2.00) | 37.80**^b^** (±5.33) |
| **NH** | 12.07**^b^** (± 1.33) | 11.40**^bc^** (± 0.40)  1.71**^bc^** (± 0.06) | 37.73**^b^** (±1.47) |
