# Supplementary material for: Thiourea and hydrogen peroxide priming improved K+ retention and source-sink relationship for mitigating salt stress in rice
Source: Sci Rep. 2021 Feb 4;11:3000. doi: 10.1038/s41598-020-80419-6 (PMC7862675; doi:10.1038/s41598-020-80419-6)
Supplement: Supplementary file 2 — Supplementary Information 2. [file 41598_2020_80419_MOESM2_ESM.docx]

**Thiourea and hydrogen peroxide priming improved K^+^ retention and source-sink relationship for mitigating salt stress in rice**

Pandey M^1,2^, Radha Krishna P^1^, Srivastava AK^1,2^*, Suprasanna P^1,2^*

*^1^Nuclear Agriculture and Biotechnology Division, Bhabha Atomic Research Centre, Mumbai 400085, India; ^2^Homi Bhabha National Institute, Mumbai-400095, India*

***For correspondence:*** *Penna Suprasanna (*[*penna888@yahoo.com*](mailto:penna888@yahoo.com) *); Ashish Kumar Srivastava (ashishbarc@gmail.com)*

**Running title: Thiourea and hydrogen peroxide mitigate salt stress in rice**

**Supplementary Fig 1: Dose-dependent post-germination phenotyping of rice seedlings under NaCl stress conditions.** The rice seedlings were grown hydroponically for 14 d under control conditions and then subjected to variable doses of NaCl such as 0 (Control), 50, 75 and 100 mM. At 7d after treatment, average fresh weight was quantified from 30 seedlings. The data represent mean±S.E and different letters the significantly changed values (DMRT, *p* ≤ 0.05*)*.

**Supplementary Fig 2: Dose-dependent post-germination phenotyping of rice seedlings under TU and H_2_O_2_ treatments.** The rice seedlings were grown hydroponically for 14 d under control conditions and then subjected to variable doses of thiourea (A) or H_2_O_2_ (B). At 7d after treatment, average fresh weight was quantified from 30 seedlings. The data represent mean±S.E and different letters the significantly changed values (DMRT, *p* ≤ 0.05*)*.
